# Long-Term Impact of an Educational Antimicrobial Stewardship Program on Management of Patients with Hematological Diseases

**DOI:** 10.3390/antibiotics10020136

**Published:** 2021-01-30

**Authors:** Ana Belén Guisado-Gil, Manuela Aguilar-Guisado, Germán Peñalva, José Antonio Lepe, Ildefonso Espigado, Eduardo Rodríguez-Arbolí, José González-Campos, Nancy Rodríguez-Torres, María Isabel Montero-Cuadrado, José Francisco Falantes-González, Juan Luis Reguera-Ortega, María Victoria Gil-Navarro, José Molina, José-Antonio Pérez-Simón, José Miguel Cisneros

**Affiliations:** 1 Department of Infectious Diseases, Microbiology and Preventive Medicine, Infectious Diseases Research Group, Institute of Biomedicine of Seville (IBiS), University of Seville/CSIC/University Hospital Virgen del Rocio, 41013 Seville, Spain; anaguigil@gmail.com (A.B.G.-G.); german.penalva@gmail.com (G.P.); josea.lepe.sspa@juntadeandalucia.es (J.A.L.); josemolinagb@gmail.com (J.M.); jmcisnerosh@gmail.com (J.M.C.); 2 Department of Pharmacy, University Hospital Virgen del Rocio, 41013 Seville, Spain; mariav.gil.sspa@juntadeandalucia.es; 3 Department of Hematology, Institute of Biomedicine of Seville (IBiS/CSIC/CIBERONC), University Hospital Virgen del Rocio, University of Seville, 41013 Seville, Spain; ildefonso.espigado.sspa@juntadeandalucia.es (I.E.); edurodarb@gmail.com (E.R.-A.); jose.gonzalez.sspa@juntadeandalucia.es (J.G.-C.); nanarotor@hotmail.com (N.R.-T.); mariai.montero.sspa@juntadeandalucia.es (M.I.M.-C.); josef.falantes.sspa@juntadeandalucia.es (J.F.F.-G.); juanlu_jlr@hotmail.com (J.L.R.-O.); josea.perez.simon.sspa@juntadeandalucia.es (J.-A.P.-S.)

**Keywords:** antimicrobial stewardship, anti-infective agents, bacteremia, candidemia, hematologic diseases

## Abstract

Antimicrobial stewardship programs (ASPs) in hematological patients are especially relevant. However, information about ASPs in this population is scarce. For 11 years, we quarterly assessed antimicrobial consumption and incidence and death rates of multidrug-resistant (MDR) bloodstream infections (BSI) in the hematology Department. Healthcare activity indicators were also monitored yearly. We performed an interrupted time-series analysis. Antimicrobials showed a sustained reduction with a relative effect of −62.3% (95% CI −84.5 to −40.1) nine years after the inception of the ASP, being especially relevant for antifungals (relative effect −80.4%, −90.9 to −69.9), quinolones (relative effect −85.0%, −102.0 to −68.1), and carbapenems (relative effect −68.8%, −126.0 to −10.6). Incidence density of MDR BSI remained low and stable (mean 1.10 vs. 0.82 episodes per 1000 occupied bed days for the pre-intervention and the ASP period, respectively) with a quarterly percentage of change of −0.3% (95% CI −2.0 to 1.4). Early and late mortality of MDR BSI presented a steady trend (quarterly percentage of change −0.7%, 95% CI −1.7 to 0.3 and −0.6%, 95% CI −1.5 to 0.3, respectively). Volume and complexity of healthcare activity increased over the years. The ASP effectively achieved long-term reductions in antimicrobial consumption and improvements in the prescription profile, without increasing the mortality of MDR BSI.

## 1. Introduction

Antimicrobial stewardship programs (ASPs) have been identified as a valuable tool to optimize the antimicrobial use in healthcare centers, improving patient outcomes and reducing adverse events and the selection pressure related to the use of antimicrobial agents [1].

In hematological patients receiving immunosuppressive therapy, collateral damages of antimicrobial consumption, especially broad-spectrum antibiotic therapy, include the selection of multidrug-resistant (MDR) microorganisms [2], an increased propensity to fungal infections [3], and microbiota dysbiosis [4]. Although, due to these reasons the impact of ASPs in patients with hematological diseases might be especially relevant, information regarding the development of antimicrobial stewardship strategies in these patients is scarce [5,6,7].

An ASP named Institutional Program for the Optimization of Antimicrobial Treatment (PRIOAM) started in our institution in January 2011. Since then, assessments of antimicrobial use, in-hospital bacterial resistance, and mortality rates associated with nosocomial bloodstream infections (BSI) have decreased significantly [8,9]. This program covers the entire hospital and presents specific interventions focused on hematological patients.

We hypothesized that a comprehensive ASP in hematological patients could also optimize antimicrobial use, reducing the overall consumption and improving the prescription profile without increasing the incidence and mortality rates of BSI produced by MDR microorganisms. Thus, the objective of the present study was to assess the impact of the PRIOAM on antimicrobial consumption and the incidence and death rates caused by MDR BSI in hospitalized adult patients with hematological diseases.

## 2. Results

Since the inception of the ASP and as part of PRIOAM educative measures (see “Intervention” at the Materials and Methods section), a total number of 218 face-to-face structured educational interviews (EI) were performed (mean 24 ± 19 EI per year). The main reasons for inappropriate antimicrobial therapy were: an incorrect selection of the drug according to the suspected diagnosis (28.4%) or inappropriate duration (28.0%) in the case of empiric treatments, and failing to de-escalate (11.0%) in the case of targeted therapies.

Moreover, 18 clinical sessions (two per year) were performed about practical aspects of common infections in hematological malignancy patients and 45 reports were produced, including one per quarter and an additional annual report to the head of the department, on the level of attainment of pre-agreed objectives.

### 2.1. Antimicrobial Consumption

The mean consumption of all antimicrobials decreased from 148.2 ± 16.2 defined daily doses (DDD) per 100 occupied bed days (OBD) in the pre-intervention period to 112.0 ± 21.7 DDD per 100 OBD in the ASP period (*p* < 0.001). Detailed data from the pre-post analysis are included in the Supplementary material (Table S1).

**Figure 1:**
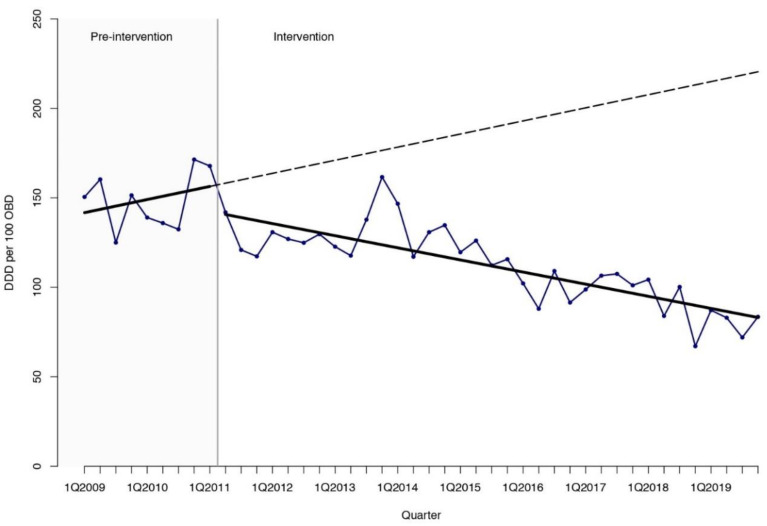
Interrupted time-series analysis of the trends in antimicrobial consumption (antibacterials for systemic use and antifungals) observed before and after the implementation of the antimicrobial stewardship program. Solid lines show the observed trend during the pre-intervention and intervention periods. Dashed lines show the expected trend after the intervention according to the pre-intervention values. DDD, defined daily doses. OBD, occupied bed days. Q, quarter.

**Figure 2:**
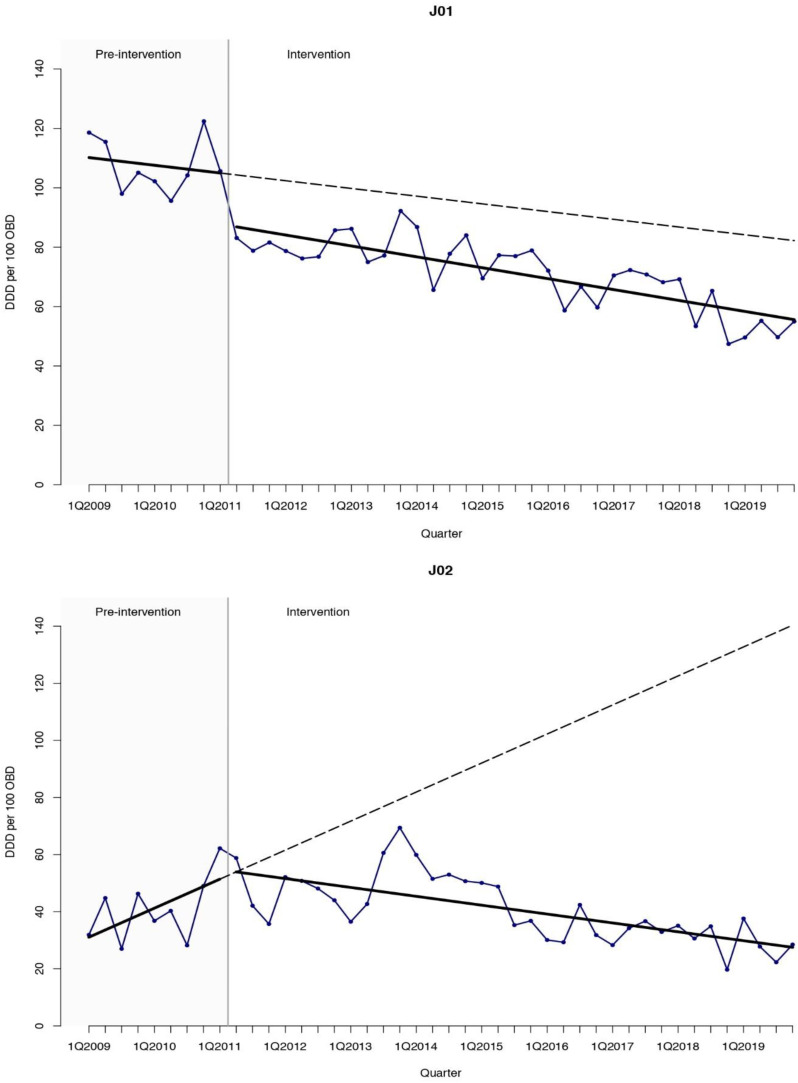
Interrupted time-series analysis of the trends in consumption for antibacterials for systemic use (**J01**) and antifungals (**J02**) observed before and after the implementation of the antimicrobial stewardship program. Solid lines show the observed trend during the pre-intervention and intervention periods. Dashed lines show the expected trend after the intervention according to the pre-intervention values. DDD, defined daily doses. OBD, occupied bed days. Q, quarter.

**Figure 3:**
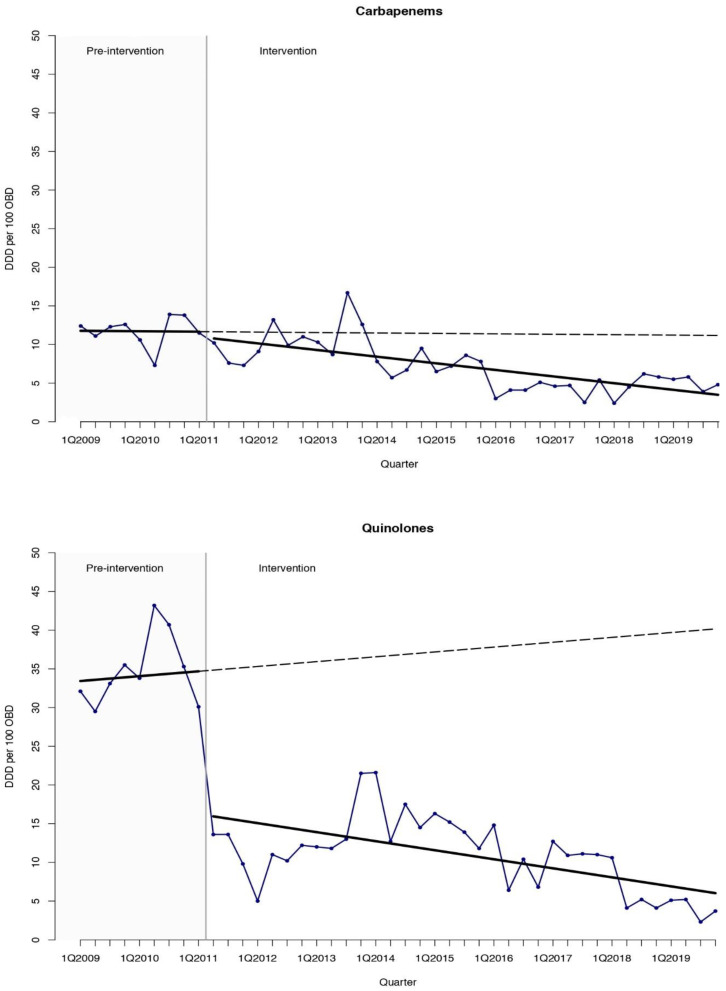
Interrupted time-series analysis of the trends in consumption for carbapenems and quinolones observed before and after the implementation of the antimicrobial stewardship program. Solid lines show the observed trend during the pre-intervention and intervention periods. Dashed lines show the expected trend after the intervention according to the pre-intervention values. DDD, defined daily doses. OBD, occupied bed days. Q, quarter.

The interrupted time-series (ITS) analysis (Table 1, Figure 1, Figure 2 and Figure 3) showed a sustained reduction in favor of the intervention with a relative effect of −62.3% (95% confidence interval [CI] −84.5 to −40.1) nine years after the inception of the ASP, when compared with the expected antimicrobial consumption based on the pre-intervention trend. As for antibiotics, a prompt change in the level after the inception of the ASP of −17.22 DDD per 100 OBD (95% CI −29.17 to −5.28) was found. Regarding antifungal consumption, a decreasing trend with a change in slope of −3.32 DDD per 100 OBD (95% CI −6.04 to −0.60) and a relative effect −80.4% (95% CI −90.9 to −69.9) was obtained with the intervention. Quinolones were the agents that showed the highest reduction with a change in the level of −18.45 DDD per 100 OBD (95% CI −25.29 to −11.62) after the start of the intervention that led to a relative effect of −85.0% (95% CI −102.0 to −68.1) at the end of the study period. Broad-spectrum antibiotics such as carbapenems and glycopeptides presented significant relative effects of −68.8% (95% CI −126.0 to −10.6) and −70.5% (95% CI −138.9 to −2.1), respectively, compared with the expected consumption based on the pre-intervention trend. The global trend is described in Table S2.

### 2.2. Clinical Outcomes

For the entire study period, the most common gram-negative microorganism causing BSI was non-extended-spectrum beta-lactamase (ESBL) producing *Escherichia coli* (48.1%). MDR gram-negative bacteria and *Candida* spp. caused 14.4% and 5.6% of BSI that were monitored, respectively (Table S3). BSI produced by MDR *Pseudomonas aeruginosa* and *Candida* spp. were responsible for the highest values of early and late mortality rates (Table S4).

For incidence density (ID) and mortality rate, the ITS analysis is shown in Table 2. The pre-post analysis and the trend analysis can be found in Tables S5 and S6, respectively.

The ID of BSI caused by MDR organisms, which kept low during the entire study period, remained stable (mean incidence 1.11 episodes per 1000 OBD for the pre-intervention period and 0.82 episodes per 1000 OBD for the ASP period) with a quarterly percentage change (QPC) of −0.3% (95% CI −2.0 to 1.4, *p* = 0.709). Early and late mortality of MDR BSI presented a steady trend with a QPC of −0.7% (95% CI −1.7 to 0.3, *p* = 0.154) and −0.6% (95% CI −1.5 to 0.3, *p* = 0.201), respectively.

### 2.3. Changes in Healthcare during the Study Period

Activity indicators related to the volume and complexity of the hematology department such as the number of blood cultures per 1000 OBD, total admissions, OBD, and the number of allogeneic hematopoietic stem-cell transplantation (HSCT) increased during the study period. Other indicators of the department’s activity remained stable (Table 3).

## 3. Discussion

The results of our study show that an education-based ASP in the hematology department was able to achieve long-term reductions in overall antimicrobial consumption and improvements in the prescription profile, especially relevant in broad-spectrum antibiotics such as carbapenems, quinolones, and antifungals, without increasing the mortality rates and maintaining a low incidence of MDR BSI. This positive impact was observed in a tertiary care hospital where infectious diseases consultation (IDC) was performed for more than 25 years and up to the PRIOAM implementation. To the best of our knowledge, this is the first study proving nine years’ data on the benefits of ASPs in the setting of hematological patients.

Very few previous studies have evaluated the effect of ASPs on antimicrobial consumption in hematological patients with most of them limited by sample size, study period (<2 years before or after intervention), and the absence of data about specific groups of antibiotics. One of the most rigorous is the study performed by So et al. [10] in leukemia units with audit and feedback as the core measures of the ASP. In contrast to our results, the intervention was associated with a significant decrease in antibiotic use (−35.1 DDD per 100 patient-days), but no significant trend in antifungal prescription was observed during a two-year period (−4.0 DDD per 100 patient-days). Two other research works with a one-year evaluation time and including solid and hematological malignancy patients showed favorable results after the beginning of antimicrobial stewardship strategies in terms of global antibiotic consumption [11] and meropenem prescription [7].

The increase of infections caused by MDR bacteria is a major health problem worldwide [12]. This challenge also affects hematological patients [13,14,15]. However, the percentage of MDR bacteria in our center was lower than previously reported by others [15,16,17,18], and notably, remained stable throughout the study period. The low prevalence of ESBL producing *E. coli* and MDR *P. aeruginosa* and, particularly, the absence of carbapenemase-producing *Enterobacteriaceae* was especially important. The sustained reduction of the use of all-class antibiotics associated with the intervention has likely contributed to preventing the generalized increase in MDR infections described in other centers.

In our study, the early and late mortality rates from MDR BSI remained stable during the intervention, showing the absence of deleterious effects for reducing antimicrobial use in these patients. Death rates were higher for BSI caused by MDR organisms as described before [12,18]. Late mortality for ESBL producing *Enterobacteriaceae* was inferior to the 21-day mortality rate reported by Trecarichi et al. [18] for non-susceptible strains (26.2%) and comparable for MDR *P. aeruginosa* (42.4%). Despite the differences in study design, population and antimicrobial utilization due to different local treatment protocols and colonization rates by MDR bacteria, preceding results, similar to ours, illustrated the potential benefits of antimicrobial stewardship approaches. The adherence to ASP recommendation has demonstrated to be an effective and safe strategy with a 64% relative risk reduction in 28-day mortality [19] and a significant decrease in the fatality rate (from 30% to 11%) [5] both in patients with febrile neutropenia and hematological or solid tumors. In patients with hematological diseases and HSCT recipients, stopping antimicrobial therapy early did not significantly increase the incidence of fever relapse and positive blood cultures or the mortality rate, with the advantage of the reduction in the use of antibiotics [20,21,22].

The results of the current study are even more remarkable if it is taken into consideration that most indicators related to the volume and complexity of the activity at the hematology department increased considerably during the study period. A fact that, in general, is related to a higher frequency of infectious complications and, as a consequence, higher consumption of antibiotics. Only the number of patients diagnosed with acute myeloid leukemia (AML), the length of hospital stay, and the transplant-related mortality within the first 100 days after allogeneic HSCT from human leukocyte antigen (HLA)-identical siblings remained stable. The monitoring of changes in healthcare as an internal control as well as the largest period of study, spanning 11 years in total, are some of the strengths of this work. Additionally, the employment of ITS analyses, the preferential method to assess the impact of health interventions over time [23], and the consistent results throughout the variables evaluated, support a potential causality relation between the ASP implementation and the progressive reduction in the antimicrobial pressure. The stable trend in the mortality by MDR BSI supports the safety of the intervention.

PRIOAM’s methods diverge from those ASP performed previously in patients with onco-hematological diseases in which educational initiatives were not incorporated as the core element of the program [11], or they were based on a sole recommendation (de-escalation, discontinuation, antibiotic cycling, etc.) [6,20,21] and/or a specific diagnosis [10], commonly febrile neutropenia [17,19,20,21]. The educational nature combined with real-time intervention(s) and the inclusion of patients with all types of hematological diseases comprise a differentiating feature of this work.

In patients with hematological diseases, post-chemotherapy febrile neutropenia was one of the most frequent infectious syndromes requiring antimicrobial courses. In this sense, the contribution of the results of the How Long clinical trial [24], led by investigators from our institution, to change the clinical practice and to decrease antibiotic overpressure in hematological patients was considerable. According to the main findings, in high-risk patients with hematological malignancies and febrile neutropenia, empirical antimicrobial therapy can be safely discontinued after 72 h of apyrexia and clinical recovery irrespective of their neutrophil count. It reinforced the previously published recommendation from the 4th European Conference on Infections in Leukaemia (ECIL-4) about empirical treatment of febrile neutropenia [25].

In our center, quinolones were agents commonly selected as an empirical combination therapy in patients with febrile neutropenia, especially in those with a suspected respiratory infection. Quinolones showed the highest reduction after the start of the intervention and the greatest decrease in the relative effect at the end of the study period. It could be explained by the fact that the ASP guidelines highly recommended the withdrawal of combination therapy 48 h after the start if an infection was not confirmed, or if it was presented and narrower spectrum antibiotics could be employed instead of quinolones. The implementation of this recommendation through the ASP has likely contributed to achieving this result.

However, for this study, some limitations should be noted. First, the study design is not exempt from the possibility of ecologic bias, and, consequently, we could not unequivocally associate the results of incidence and mortality of MDR organisms to the ASP implementation. Although the volume and complexity of the activity in the hematology department were monitored, other potential confounding factors such as those related to patients and the center could possibly interfere with the outcomes. In addition, the single-center design limits the external validity of our results and makes it necessary to confirm the reproducibility of the findings in different settings. Second, the close relationship between the IDC in the pre-intervention period and the ASP made it difficult to elucidate the precise weight of each one on the outcomes achieved. Nonetheless, regarding the use of antimicrobials, the stable trends during the pre-intervention period suggest that the implementation of the ASP was necessary to achieve the goals. The sole IDC was insufficient to promote a change in the entire department, as reported in previous studies [26]. Finally, non-MDR BSI, invasive candidiasis (other than candidemia) and aspergillosis have not been examined in this study. The decreasing trend in overall antimicrobial consumption, including voriconazole and liposomal amphotericin B as common treatments for invasive infections caused by molds [27], suggests, at least, a steady frequency of these infections.

## 4. Materials and Methods

### 4.1. Study Design and Period

A quasi-experimental before-after study of ITS was performed. The PRIOAM implementation started in January 2011, and, since then, data were prospectively registered for a nine-year period. For the ITS analyses, the study period spanned 44 quarters (11 years) from January 2009 to December 2019.

### 4.2. Setting

The program was performed at the 39-bed hematology department of the University Hospital Virgen del Rocio (Seville, Spain), which is a teaching hospital providing a tertiary-care service in Southwest Spain. The hospital, with 1177 beds and 72 intensive care unit beds, is a referral-center for solid-organ and HSCT. Adult patients (aged ≥ 18 years) receiving treatment for hematological malignancies or undergoing HSCT are treated in this unit. Throughout the last nine years, the hematology department has admitted a mean number of 1134 adult patients per year and has performed a mean of 108 autologous and allogeneic HSCT per year in adults.

### 4.3. Intervention

The PRIOAM’s methods and global outcomes have already been published [8,9]. In brief, it comprises a bundle of educational strategies performed by a multidisciplinary team including infectious diseases physicians, microbiologists, pharmacists, intensive care physicians, pediatricians, and preventivists. The core elements of PRIOAM are summarized in the Supplementary material (Figure S1).

Because most EI were performed when a potentially inappropriate prescription was detected (i.e., use of carbapenems or combination therapy for >48 h, antibiotic duration >7 days or targeted therapies), the main messages tackled in EI were: early identification and management of severe infections, interpretation of microbiologic results, de-escalation and sequential oral treatments whenever possible, diversification of antimicrobial prescriptions, and training in the optimal duration of antimicrobial courses. The form employed for EI is included as Figure S2. No other interventions concerning antimicrobial use (i.e., antimicrobial policies, restrictions, etc.) were performed during the study period. The infection control program in the hematology ward consisted of the isolation in high-efficiency particulate air (HEPA) filters conditioned rooms of neutropenic and HSCT patients, and contact isolation of patients with MDR bacteria or respiratory viruses recovered from clinical samples. Local guidelines for antifungal prophylaxis did not change substantially during the intervention period. No additional measures were implemented regarding infection prevention, and antibiotic prophylaxis was not recommended for hematological patients in our center since 2005.

Before the start of the PRIOAM, a stable IDC program was running at the hematology department, consisting of bedside advice for the management of complex infections, quick report of all BSI, the production and application of local guidelines, updated every two years, for the prevention, diagnosis, and management of infections, and surveillance and analyses of MDR outbreaks. The usual IDC led the implementation of antimicrobial stewardship tasks in the hematology department.

### 4.4. Study Measures

Antimicrobial use was evaluated through quarterly measures of the antibiotic consumption of the Anatomical Therapeutical Chemical (ATC) group J01 (antibacterials for systemic use) and antifungals ATC group J02. Data about antimicrobial consumption were automatically generated by the electronic prescribing system, which provided information about the units (capsules, injection vials, etc.) used by each department. Consumption was calculated as DDD per 100 OBD, according to the ATC Classification methodology and the 2019 World Health Organization DDD values [28]. Because no DDD was suggested for liposomal amphotericin B, we considered the 210 mg dose as the unit.

For the study period, BSI caused by the most relevant microorganisms in patients with hematological diseases (*E. coli*, *Klebsiella pneumoniae*, *P. aeruginosa*, and *Candida* spp.) were registered. The effect of the intervention on the number of BSI produced by MDR microorganisms (ESBL-producing *Enterobacteriaceae*, MDR *P. aeruginosa*, and *Candida* spp.) was monitored quarterly and presented as ID per 1000 OBD. The German Society for Hygiene and Microbiology criteria [29] was taken into account for MDR categorization. The analysis of antibiotic susceptibility and resistance mechanisms was performed following the European Committee on Antimicrobial Susceptibility Testing (EUCAST) criteria [30,31].

The effect on the mortality rates was assessed as the all-cause crude death rate [9,32] (deaths per 1000 OBD per quarter) on day +7 (early mortality) and +30 (late mortality) after the diagnosis of BSI. Patients dying in less than 24 h after blood sample collection were not considered for the mortality analysis, as previously proposed [26,33,34], for a better selection of patients benefitting from the intervention targeting an optimized use of antimicrobials.

To analyze the effect of changes in the hematology department during the 11-year study period, we monitored yearly indicators related to the volume and complexity of the activity at the department that may influence the antimicrobial use, such as the number of blood cultures per 1000 OBD, new patients diagnosed with AML, admissions, and OBD, as well as the mean length of stay. We also monitored the number of allogeneic HSCT and the transplantation-related mortality within the first 100 days after allogeneic HSCT from HLA-identical siblings.

Because presentation, dissemination, and introduction activities of PRIOAM took place in the different departments of the hospital from January to 31 March 2011, we considered 1 April as the beginning of the intervention period for the analysis.

### 4.5. Statistical Analysis

For descriptive aims, categorical variables were presented as frequency distribution and percentages, and continuous variables were presented as means ± standard deviations (SD). The Student’s *t*-test or the Mann-Whitney U test were employed for univariate pre-post analyses, after checking for normality using the Kolmogorov-Smirnov test.

To assess the effect of the ASP, an ITS analysis was performed to estimate changes in the level and trends before and after the inception of the program. We used a generalized least squares regression approach accounting for autocorrelation by autoregressive moving-average (ARMA) models. The final model selection for each variable was based on the Akaike Information Criterion with validation of the autocorrelation structures by likelihood ratio tests [35]. The long-term effect attributable to the ASP for each outcome was estimated by calculating the relative effect, as the percentage difference between the values of the expected pre-intervention trend and the modeled trend at the end of the study. Alternatively, a joinpoint regression analysis was conducted to explore the trends of the time-series [36], calculating the QPC during the 11-year study period by using the Joinpoint software modeling annual percentage change calculation to our log-transformed quarterly data with autocorrelated error models.

Confidence intervals or *p-*values (*p*) were included to show statistical significance. Differences were considered statistically significant at *p* < 0.05 (2-tailed tests). Statistical analyses were performed with IBM SPSS Statistics software v. 23.0, R software v. 3.5.2 and Joinpoint Regression Program v. 4.6.0.0.

### 4.6. Ethics Approval

The study was conducted in accordance with the Declaration of Helsinki, and the protocol was approved by the Ethics Committee of the University Hospital Virgen del Rocio (Project identification code: PI-0361-2010).

## 5. Conclusions

These results allow us to state that an education-based ASP contributed significantly to the decreasing trend in the use of antimicrobials and, possibly, to maintain the low incidence of MDR BSI despite the increase in the volume and complexity of the activity at the hematology department over the study period. Death rates of BSI caused by MDR organisms were stable, showing that these interventions are safe in this vulnerable population.

## Figures and Tables

**Table 1:** Interrupted time-series analysis of changes in trends of antimicrobial consumption.

Outcomes	Pre-Intervention Trend	Change in Level ^a^	Change in Trend ^b^	Relative Effect ^c^ %
**Total J01+J02**	1.83 (−2.14 to 5.80)	−13.98 (−35.65 to 7.69)	−3.52 (−7.57 to 0.52)	−62.3 (−84.5 to −40.1)
**Total antibiotics (J01)**	−0.65 (−2.84 to 1.54)	−17.22 (−29.17 to −5.28)	−0.27 (−2.49 to 1.95)	−32.4 (−99.2 to 34.5)
**Total antifungals (J02)**	2.54 (−0.12 to 5.20)	3.31 (−11.12 to 17.74)	−3.32 (−6.04 to −0.60)	−80.4 (−90.9 to −69.9)
**Carbapenems**	−0.01 (−0.69 to 0.66)	−0.67 (−4.33 to 2.99)	−0.20 (−0.89 to 0.49)	−68.8 (−126.0 to −10.6)
**Piperacillin-tazobactam**	0.63 (−0.25 to 1.51)	7.78 (3.30 to 12.27)	−0.86 (−1.78 to 0.07)	−67.3 (−96.9 to −38.6)
**Antipseudomonal** **cephalosporins**	−0.55 (−1.57 to 0.47)	−10.79 (−16.18 to −5.41)	0.77 (−0.29 to 1.82)	105.1 (−195.6 to 405.8)
**Quinolones**	0.16 (−1.16 to 1.47)	−18.45 (−25.29 to −11.62)	−0.45 (−1.82 to 0.93)	−85.0 (−102.0 to −68.1)
**Amikacin**	−0.03 (−0.46 to 0.39)	1.68 (−0.51 to 3.87)	−0.05 (−0.48 to 0.39)	0.1 (−410.8 to 413.6)
**Glycopeptides**	0.01 (−0.55 to 0.56)	0.68 (−2.27 to 3.62)	−0.17 (−0.74 to 0.40)	−70.5 (−138.9 to −2.1)

Data are presented as quarterly defined daily doses per 100 occupied bed days with a 95% confidence interval, unless otherwise specified. ^a^ Increase or decrease in the first quarter after the start of the antimicrobial stewardship program (ASP) period with respect to the expected value. ^b^ Change in slope for the ASP period. ^c^ Percentage difference between the expected value according to the pre-intervention trend and the trend nine years after the start of the ASP.

**Table 2:** Interrupted time-series analysis of changes in trends of incidence and mortality rate of multidrug-resistant bloodstream infections.

Outcomes	Pre-Intervention Trend	Change in Level ^a^	Change in Trend ^b^	Relative Effect ^c^ %
**Incidence** **density**	−0.09 (−0.25 to 0.07)	−0.11 (−1.00 to 0.77)	0.10 (−0.06 to 0.26)	98.9 (−301.4 to 499.2)
**Early mortality**	0.009 (−0.03 to 0.05)	0.06 (−0.14 to 0.26)	−0.01 (−0.05 to 0.03)	−72.1 (−147.8 to 3.5)
**Late mortality**	0.01 (−0.06 to 0.08)	−0.03 (−0.42 to 0.36)	−0.005 (−0.08 to 0.07)	−35.55 (−346.9 to 275.8)

Data are presented as quarterly incidence density and all-cause crude death rate per 1000 occupied bed days with a 95% confidence interval, unless otherwise specified. ^a^ Increase or decrease in the first quarter after the start of the antimicrobial stewardship program (ASP) period with respect to the expected value. ^b^ Change in slope for the ASP period. ^c^ Percentage difference between the expected value according to the pre-intervention trend and the trend nine years after the start of the ASP.

**Table 3:** Indicators related to the volume and complexity of the activity at the hematology department.

Outcomes	2009	2010	2011	2012	2013	2014	2015	2016	2017	2018	2019	APC (95% CI)
**Blood cultures per 1000 OBD**	72	71	59	71	100	121	92	100	100	133	102	6.014 (2.348 to 9.811)
**AML**	21	33	35	47	35	37	35	29	43	35	37	4.400 (−6.186 to 16.279)
**Admissions**	1005	1055	1081	946	1052	1148	1169	1120	1133	1290	1265	2.336 (1.253 to 3.430)
**OBD**	8966	9128	10,616	10,463	10,343	10,620	10,840	11,135	11,753	11,719	14,463	3.540 (2.358 to 4.735)
**Length of stay, mean**	16	15	18	17	17	16	16	16	17	14	16	−0.843 (−1.908 to 0.235)
**Allogeneic HSCT**	19	20	33	40	47	55	56	58	44	43	47	8.609 (4.436 to 12.948)
**HSCT−related mortality, %**	5.3	5.0	0	0	6.4	1.8	7.1	1.7	0	2.3	2.1	−7.007 (−16.347 to 3.376)

For each year, data are presented as the number of events, unless otherwise specified. In the last column, the annual percentage of change (APC) obtained from joinpoint regression analysis with a 95% confidence interval (CI) is included. OBD, occupied bed days. AML, acute myeloid leukemia. HSCT-related mortality, hematopoietic stem-cell transplantation (HSCT)-related mortality within the first 100 days after allogeneic HSCT from human leukocyte antigen (HLA)-identical siblings.

## Data Availability

The data presented in this study are available on request from the corresponding author.

## Supplementary Materials

The following are available online at https://www.mdpi.com/2079-6382/10/2/136/s1. Table S1: Differences between the pre-intervention period and the antimicrobial stewardship program period regarding pre-post analysis of antimicrobial consumption. Table S2: Trend analysis of antimicrobial consumption (2009–2019). Table S3: Frequency of most relevant gram-negative microorganisms and Candida spp. as causative agents of bloodstream infections (2009–2019). Table S4: Mortality of patients with the most relevant gram-negative microorganisms and Candida spp. causing bloodstream infections (2009–2019). Table S5: Differences between the pre-intervention period and the antimicrobial stewardship program period regarding pre-post analysis of incidence and mortality rate of multidrug-resistant bloodstream infections. Table S6: Trend analysis of the incidence and mortality rate of multidrug-resistant bloodstream infections (2009–2019). Figure S1: Description of the core elements of PRIOAM. Figure S2: Form for PRIOAM educational interviews.
