# Supplementary material for: Long-Term Impact of an Educational Antimicrobial Stewardship Program on Management of Patients with Hematological Diseases
Source: Antibiotics (Basel). 2021 Jan 30;10(2):136. doi: 10.3390/antibiotics10020136 (PMC7911074; doi:10.3390/antibiotics10020136)
Supplement: Supplementary file 1 [file antibiotics-10-00136-s001.pdf]

antibiotics

Supplementary material

Table S1. Differences between the pre-intervention period and the antimicrobial stewardship program period regarding pre-post analysis of antimicrobial consumption.

Outcomes	Pre-Intervention period	ASP period	*P* value
**Total J01+J02**	148.2±16.2	112.0±21.7	<0.001
**Antibiotics (J01)**	107.5±9.3	71.2±11.7	<0.001
**Antifungals (J02)**	40.8±11.3	40.8±11.7	0.954
**Carbapenems**	11.7±2.0	7.1±3.2	<0.001
**Piperacillin-tazobactam**	6.9±1.6	14.9±4.0	<0.001
**Antipseudomonal cephalosporins**	15.9±3.3	6.9±4.0	<0.001
**Quinolones**	34.8±4.6	10.9±4.8	<0.001
**Amikacin**	5.1±0.8	5.5±1.7	0.782
**Glycopeptides**	7.3±1.9	4.9±2.4	0.009

Data are presented as mean±standard deviation of quarterly defined daily doses per 100 occupied bed days. *P* values represent the results from Student's t-test or Mann-Whitney U, according to the data distribution. ASP, antimicrobial stewardship program.

Table S2. Trend analysis of antimicrobial consumption (2009-2019).

Outcomes	QPC (%)	95% CI	*P* value
**Total J01+J02**	−1.455	(−2.011 to −0.896)	<0.001
**Antibiotics (J01)**	−1.622	(−1.925 to −1.317)	<0.001
**Antifungals (J02)**	−0.857	(−1.813 to 0.108)	0.082
**Carbapenems**	−2.787	(−3.666 to −1.899)	<0.001
**Piperacillin-tazobactam**	1.220	(−0.201 to 2.662)	0.093
**Antipseudomonal cephalosporins**	−1.301	(−4.205 to 1.690)	0.390
**Quinolones**	−4.381	(−5.963 to −2.773)	<0.001
**Amikacin**	−0.605	(−1.911 to 0.718)	0.368
**Glycopeptides**	−2.621	(−3.614 to −1.618)	<0.001

Data are presented as quarterly defined daily doses per 100 occupied bed days. QPC, quarterly percentage change. CI, confidence interval.

Table S3. Frequency of most relevant gram-negative microorganisms and *Candida* spp. as causative agents of bloodstream infections (2009–2019).

Microorganism	Number (%) N = 522
**Gram-negative microorganisms**	493
Escherichia coli	273 (55.4)
ESBL *E. coli*	36 (13.2)
Klebsiella pneumoniae	116 (23.5)
ESBL *K. pneumoniae*	26 (22.4)
Pseudomonas aeruginosa	82 (16.6)
MDR *P. aeruginosa*	13 (15.9)
***Candida* spp.**	29

ESBL, extended-spectrum β-lactamase. MDR, multidrug-resistant.

Table S4. Mortality of patients with the most relevant gram-negative microorganisms and *Candida* spp. causing bloodstream infections (2009-2019).

Microorganism	Number of patients	Number of deaths on day +7 (%)	Number of deaths on day +30 (%)
**MDR microorganisms**	104	13 (12.5)	29 (27.9)
ESBL *Escherichia coli*	36	2 (5.6)	6 (16.7)
ESBL *Klebsiella pneumoniae*	26	2 (7.7)	4 (15.4)
MDR *Pseudomonas aeruginosa*	13	3 (23.1)	6 (46.2)
Candida spp.	29	6 (20.7)	13 (44.8)
**Non-MDR microorganisms**	396	23 (5.8)	56 (14.1)
Escherichia coli	237	13 (5.5)	35 (14.8)
Klebsiella pneumoniae	90	6 (6.7)	13 (14.4)
Pseudomonas aeruginosa	69	4 (5.8)	8 (11.6)

MDR, multidrug-resistant. ESBL, extended-spectrum β-lactamase.

Table S5. Differences between the pre-intervention period and the antimicrobial stewardship program period regarding pre-post analysis of incidence and mortality rate of multidrug-resistant bloodstream infections.

Outcomes	Pre-Intervention period	ASP period	*P* value
**Incidence density**	1.11±0.76	0.82±0.61	0.210
**Early mortality**	0.05±0.16	0.12±0.19	0.308
**Late mortality**	0.15±0.32	0.26±0.27	0.226

Data are presented as mean±standard deviation of quarterly incidence density and all-cause crude death rate on day +7 (early mortality) and +30 (late mortality) per 1000 occupied bed days. *P* values represent the results from Student's t-test or Mann-Whitney U, according to the data distribution. ASP, antimicrobial stewardship program.

Table S6. Trend analysis of the incidence and mortality rate of multidrug-resistant bloodstream infections (2009-2019).

Outcomes	QPC (%)	95% CI	*P* value
**Incidence density**	−0.325	(−2.049 to 1.431)	0.709
**Early mortality**	−0.697	(−1.657 to 0.272)	0.154
**Late mortality**	−0.608	(−1.545 to 0.337)	0.201

Data are presented as quarterly incidence density and all-cause crude death rate on day +7 (early mortality) and +30 (late mortality) per 1000 occupied bed days. QPC, quarterly percentage change. CI, confidence interval.

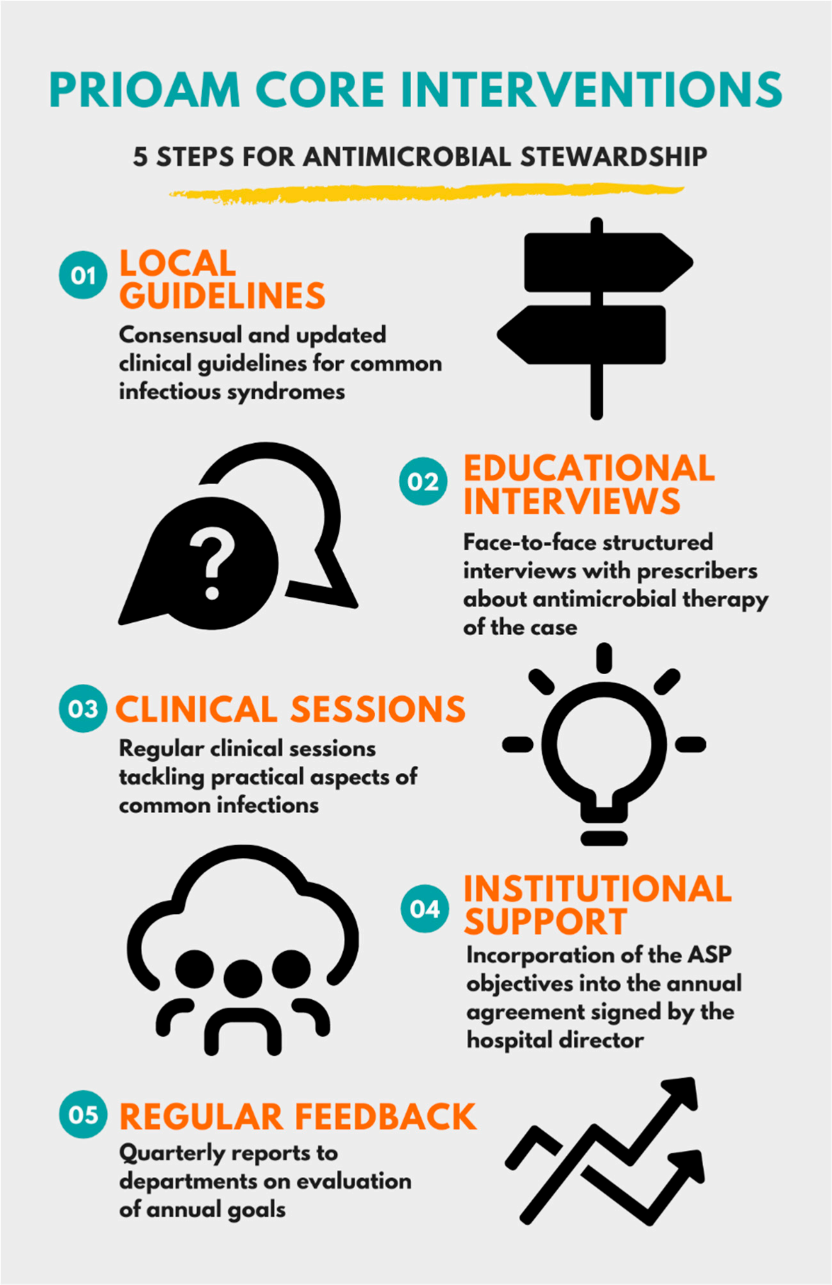

Figure S1. Description of the core elements of PRIOAM.

Date of interview: Centre
Clinical department Episode number
Advisor: Prescriber
Antimicrobial agent(s)
Clinical indication:

- ☐ Perioperative prophylaxis
- ☐ Diagnosis without microbiological confirmation
- ☐ Diagnosis with microbiological confirmation

Describe

☐ **Perioperative prophylaxis**

1. Was prophylaxis indicated? ☐ Yes ☐ No
2. Was the chosen agent appropriate? ☐ Yes ☐ No
3. Was the administration timing appropriate? ☐ Yes ☐ No
4. Was the total number of doses appropriate? ☐ Yes ☐ No

☐ **Empirical antimicrobial treatment**

1. Was empirical treatment initiation indicated? ☐ Yes ☐ No
2. Was the timing of treatment initiation appropriate? ☐ Yes ☐ No
3. Were microbiological samples collected?
   It was not indicated:
   ☐ Not performed
   It was indicated:
   ☐ Performed
   ☐ Not performed or incorrectly performed
4. Was the chosen agent appropriate? ☐ Yes ☐ No
5. Was the dosing appropriate? ☐ Yes ☐ No
6. Was the way of administration appropriate? ☐ Yes ☐ No
7. If other therapeutic measures were indicated, were they performed correctly?
   They were not indicated and not performed ☐
   They were indicated and correctly performed ☐
   They were indicated, but not correctly performed ☐
8. Is the planned treatment duration appropriate? ☐ Yes ☐ No

☐ **Targeted antimicrobial treatment**

1. Was antimicrobial treatment indicated? ☐ Yes ☐ No
2. Was the timing of the treatment initiation appropriate? ☐ Yes ☐ No
3. Was the interpretation of the microbiological results correct? ☐ Yes ☐ No
4. Was the chosen agent appropriate? ☐ Yes ☐ No
5. Was the chosen agent the most appropriate? ☐ Yes ☐ No
6. Was the dosing appropriate? ☐ Yes ☐ No
7. Was the way of administration appropriate? ☐ Yes ☐ No
8. If other therapeutic measures were indicated, were they performed correctly?
   They were not indicated and not performed ☐
   They were indicated and correctly performed ☐
   They were indicated, but not correctly performed ☐

1\. Is the planned treatment duration appropriate? ☐ Yes ☐ No

Figure S2. Form for PRIOAM educational interviews.
